# A Compressive Review about Taxol^®^: History and Future Challenges

**DOI:** 10.3390/molecules25245986

**Published:** 2020-12-17

**Authors:** Julia Gallego-Jara, Gema Lozano-Terol, Rosa Alba Sola-Martínez, Manuel Cánovas-Díaz, Teresa de Diego Puente

**Affiliations:** Department of Biochemistry and Molecular Biology (B) and Immunology, Faculty of Chemistry, University of Murcia, Campus de Espinardo, Regional Campus of International Excellence “Campus Mare Nostrum”, P.O. Box 4021, 30100 Murcia, Spain; gema.lozano@um.es (G.-L.T.); rosaalba.sola@um.es (R.A.S.-M.); mcanovas@um.es (M.C.-D.); tdp@um.es (T.d.D.P.)

**Keywords:** drug, cancer, cell death, biotechnology, antitumor agent

## Abstract

Taxol^®^, which is also known as paclitaxel, is a chemotherapeutic agent widely used to treat different cancers. Since the discovery of its antitumoral activity, Taxol^®^ has been used to treat over one million patients, making it one of the most widely employed antitumoral drugs. Taxol^®^ was the first microtubule targeting agent described in the literature, with its main mechanism of action consisting of the disruption of microtubule dynamics, thus inducing mitotic arrest and cell death. However, secondary mechanisms for achieving apoptosis have also been demonstrated. Despite its wide use, Taxol^®^ has certain disadvantages. The main challenges facing Taxol^®^ are the need to find an environmentally sustainable production method based on the use of microorganisms, increase its bioavailability without exerting adverse effects on the health of patients and minimize the resistance presented by a high percentage of cells treated with paclitaxel. This review details, in a succinct manner, the main aspects of this important drug, from its discovery to the present day. We highlight the main challenges that must be faced in the coming years, in order to increase the effectiveness of Taxol^®^ as an anticancer agent.

## 1. Introduction

Taxol^®^, which has the generic name paclitaxel (PTX), is a secondary metabolite produced by *Taxus* sp. and, to a lesser extent, by *Coniferales Cephalotaxus*, *Podocarpus gracilior*, or *Corylus avellana* [1,2,3]. The first identification of PTX was carried out by Dr. Jonathan L. Hartwell (National Cancer Institute, NCI) in the 1960s, when a screening program for antitumor agents in the plant kingdom was carried out by the NCI and the U.S. Department of Agriculture, identifying PTX from *Taxus brevifolia* (*T. brevifolia*) as a potent anticancer drug [4]. The structure of PTX was published in 1971 and clinical trials began in 1984 [5,6]. The high demand for PTX due to these clinical trials led to a severe depletion of *T. brevifolia*. Furthermore, the cost of manufacturing PTX from *T. brevifolia* was 10 times the budget available for the trials. These events led to a race to develop a chemical synthesis route for PTX, which was finally produced in 1994 [7]. However, the method was too complex and expensive, so the NCI decided to leave the work developed until then in the hands of a private company, not being able to bear the costs. The chosen company was Bristol-Myers Squibb (BMS), despite complaints about a private company acquiring the rights to this drug. BMS renamed PTX under the brand name Taxol^®^ and left the term paclitaxel as the generic name. Taxol^®^ global sales have been in the millions since it began to be marketed, reaching 1.5 billion dollars in 2000. However, since then, sales of Taxol^®^ have been declining rapidly to the benefit of other paclitaxel formulations, such as Abraxane^®^ [8]. The price of Taxol^®^ has also been very high. When the use of Taxol as an anti-cancer drug was approved, its price was $986/dose. However, when the BMS patent expired, the price became $150/dose for the generic version and $1000 for the Taxol^®^ version [9]. The Food and Drug Administration (FDA) and the European Medicines Agency (EMA) have approved the use of Taxol^®^ for the treatment of breast, ovarian, and lung cancer, as well as Kaposi’s sarcoma [10]. Although the response rate depends on different factors, including the type of cancer, Taxol^®^’s response rate has been approximated to be 30% for ovarian cancer and 56% for metastatic breast cancer [11,12]. Over the years, PTX, as Taxol^®^ or alternative formulations, has become one of the most-used agents for treating different types of cancers, especially breast, ovarian, non-small-cell lung carcinoma (NSCLC), Kaposi’s sarcoma, head, and leucopenia cancers. Moreover, PTX is often used as a reference to evaluate the therapeutic benefits achieved with the co-administration of another drug, for example, to simultaneously treat both stromal and tumor compartmentss in pancreatic ductal adenocarcinoma (PDAC) [13,14]. Considering this, Taxol^®^’s demand has spurred a battle over the most profitable anti-tumor domain in history [5,8,15,16].

### 1.1. Taxol^®^ Biosynthesis

Taxol^®^ is a metabolite belonging to the isoprenoid family, which is the largest natural products family. The terpenoid family is a very chemically diverse group, with members found in almost all life forms and a wide range of applications [17]. Terpenes are generally classified according to the number of isoprene molecules (C5) that form their final structure. In this sense, PTX is a diterpene (C20) formed through the condensation of four isoprene molecules. Two main terpenoid biosynthetic pathways have been identified to date: The mevalonate pathway (MVA) and the non-mevalonate pathway or methylerythritol phosphate pathway (MEP). Moreover, an alternative MEP shunt has recently been discovered [18]. The MVA pathway was discovered by Lynen and Bloch in the 1960s, who were jointly awarded the Nobel Prize for physiology in 1964. The MVA pathway involves the synthesis of isopentenyl pyrophosphate (IPP) and dimethylalyl pyrophosphate (DMAPP) from three molecules of acetyl-CoA, and occurs in animals, plants (cytosol), fungi, and archaea [19]. More recently, in the 1990s, the MEP pathway was discovered by Rohmer et al. [20,21]. MEP biosynthesis produces IPP and DMAPP through the condensation of pyruvate and glyceraldehyde 3-phosphate, and mainly occurs in eubacteria, green algae, and higher plants [22,23].

**Figure 1:**
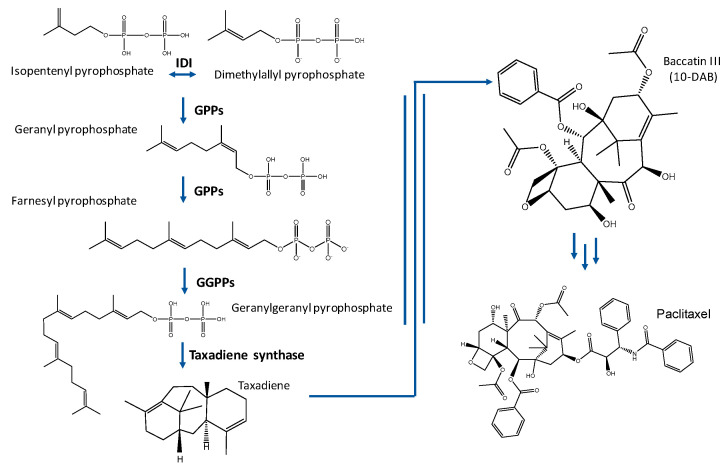
Taxol^®^ biosynthetic pathway. IDI: Isopentenyldiphosphate isomerase; GPPS: geranylpyrophosphate synthase; and GGPPS: geranylgeranylpyrophosphate synthase.

Isoprenoids are synthetized from IPP and DMAPP through the action of multiple enzymes (Figure 1). PTX biosynthesis occurring in *Taxus* sp. is complex and involves 19 distinct enzymatic steps, with eight P450-mediated oxygenation reactions [24]. One of the essential steps in the biosynthesis of Taxol^®^ is the cyclation of geranyl geranyl pyrophosphate to form taxa-4(5),11(12)-diene (taxadiene), the universal core of Taxol^®^, and its derivative compounds (taxanes). Several subsequent reactions are required to produce baccatin III, which is a late intermediate for obtaining the PTX structure [24].

### 1.2. Taxol^®^ Production

Since the discovery of the potent antitumoral properties of Taxol^®^, its worldwide demand and price have increased exponentially [8]. Large agricultural extensions of *Taxus* sp. and non-environmentally friendly extraction processes have forced the development of alternative Taxol^®^ production methods, such as chemical synthesis, the search for non-*Taxus* plant producers, endophytic fungi cultures, and the metabolic engineering of natural and heterologous hosts [6,25,26]. Since the 1990s, several complete syntheses have been published, although their complexity makes them unviable at an industrial level [7,27,28]. Shortly after elucidating the complete synthesis, semi-synthetic methods were published, starting from Taxol^®^ intermediates such as 10-deacetyl baccatin III (10-DAB), which is more abundant than Taxol^®^ in *Taxus* sp. [29]. In this way, it was possible to increase the yield of synthesis and extraction from natural sources, but production still relied on yew trees. Despite efforts made over the last 20 years to develop cheaper and more sustainable methods (which will be discussed below), Taxol^®^ extraction from trees is still the most frequently used method, which implies that, to treat a single patient with 2 g of Taxol^®^, four trees have to be felled [30].

Fermentation using genetically modified microorganisms to obtain Taxol^®^, or precursors that allow for easier chemical semi-synthesis, have positioned themselves as the most promising alternative to stop the uncontrolled felling of trees [31]. The development of molecular biology, genetic engineering, and bioinformatics in recent years has contributed to the great growth experienced by microbial biotechnology [31,32]. The production of highly valuable compounds employing genetically modified microorganisms has been considered a hopeful alternative for relieving the great disadvantages of extraction and chemical synthesis based on natural sources. Regarding the microbial production of Taxol^®^, to the best of our knowledge, a complete synthesis has not yet been achieved. However, several methods for taxadiene production have been developed. Therefore, *Escherichia coli* (*E. coli*), *Sacharomyces cerevisiae* (*S. cerevisiae*), and *Bacillus subtilis* (*B. subtillis*) have been successfully employed to produce taxadiene. In 2010, Ajikumar et al. reached a production of 1 g/L by following a two-module strategy in *E. coli*. They optimized a native upstream MEP pathway and a heterologous downstream taxadiene-forming pathway with the minimal accumulation of indole, which acts as an inhibitory compound [33]. In *S. cerevisiae*, Engels et al. achieved 8.7 mg/L through the co-expression of *T. chinensis* taxadiene synthase and *Sulfolobus acidocaldarius* geranylgeranyl diphosphate synthase [34]. More recently, a titer of 72.8 mg/L was reached by Ding et al., who selected a geranylgeranyl diphosphate synthase enzyme by a computer-aided docking strategy [35]. *B. subtilis* was used by Abdallah et al. to obtain a final concentration of 17.8 mg/L of taxadiene through the overexpression of several natural and heterologous biosynthetic enzymes [36]. Regarding the production of taxanes closer to the final structure of PTX, in 2016, Walters et al. successfully expressed heterologous P-450 oxygenases in *E. coli* to produce several oxygenated taxanes, which represents an important advance in the development of a de novo Taxol^®^ biosynthetic method [37]. Table 1 summarizes the yields achieved in the production of Taxol^®^ intermediates using microorganisms by the main works carried out to date.

Endophytic fungi have received significant attention in recent years as a means for secondary metabolite production and an alternative to microbial fermentation or chemical synthesis [38]. Fungal endophytes are organisms that colonize plants without causing (at least apparent) damage. Many of these endophytes can produce secondary metabolites which are proper to the host plant, representing a benefit for this plant [39]. Regarding Taxol^®^ production, around 200 endophytic fungi belonging to more than 40 fungal genera from several different orders have been identified as Taxol^®^ producers [30,40,41]. However, a lack of understanding about the biological physiology of endophytic fungi and the loss of Taxol^®^ productivity after multiple subcultures are the main obstacles to achieving a competitive Taxol^®^ production method [42,43,44]. Optimization of the fermentation conditions, such as the culture temperature, pH, rotation speed, carbon sources, nitrogen sources, precursors, inducers, and inhibitors, is the main focus of scientific research at present [45]. In this regard, several studies have reported Taxol^®^ production by endophytic fungi, such as *Aspergillus flavipes* (185 µg/L), *Aspergillus aculeatinus* (1.3 mg/L), *Aspergillus oryzae* (95 µg/L), and *Metarizium anisopliae* (0.16 mg/L) [46,47,48,49]. In a more recent study carried out by Kumar et al., 1.6 g/L of Taxol^®^ was yielded by employing the endophytic fungus *Aspergillus fumigatus*, which, to the best of our knowledge, comprises the highest Taxol^®^ production from an endophytic fungus [50].

### 1.3. Taxol^®^: Formulations and Bioavailability

Taxol^®^ is a tricyclic diterpenoid with a complex chemical structure involving a taxane ring (Figure 1). This taxane ring, as well as the C13 side chain, the oxetane ring, the 2′ position of the hydroxyl group, and the homochiral ester chain, are essential for its correct antitumoral activity. However, the hydroxyl group at position C-7 and acetylation of the C-10 hydroxyl group are not essential for the antitumor activity. Therefore, docetaxel, which is a synthetic analog of Taxol^®^ without the acetyl group in this position, has also shown high antitumoral activity [51].

One of the main limitations of PTX is its low solubility in water (0.1 µg/mL), which complicates its drug supply [52]. To solve this problem, the first Taxol^®^ formulation included Cremophor EL (CrEL) as a formulation vehicle, as well as absolute ethanol. CrEL is a heterogeneous non-ionic surfactant used as a vehicle for different poorly-soluble drugs. However, CrEL is not an innocuous transporter, having been associated with severe anaphylactoid hypersensitivity reactions, hyperlipidemia, abnormal lipoprotein patterns, the aggregation of erythrocytes, and peripheral neuropathy [53]. Due to the significant limitations and disadvantages of CrEL, much research has been conducted to obtain alternatives [54,55].

In recent years, the use of nano-sized materials for different purposes has led to the birth of nanomedicine [12]. Nanomaterials have been widely used for the delivery of drugs, including Taxol^®^. Since 2000, when the Taxol^®^ formulation was released, new formulations have begun to emerge from many powerful pharmaceutical companies. The great difference between them lies in the drug vehicle. Therefore, different PTX-based nanotechnological vehicles have been developed and approved, such as albumin-based nanoparticles (e.g., Abraxane^®^), polymeric lipidic nanoparticles (e.g., PICN^®^), polymeric micelles (e.g., Cynviloq^®^, Nanoxel^®^, and Paclical^®^), and liposomes (e.g., Lipusu^®^), and many others are in clinical trial phases [8,56,57,58,59,60,61,62,63,64,65] (Table 2). Abraxane^®^ has undoubtedly been the most successful and is the current leader, well above Taxol^®^ [8,62,66]. However, although the overall response rate is clearly better for Abraxane^®^ than for Taxol^®^, its response rate is only 33% (Taxol^®^’s response rate is 25%) in metastatic breast cancer patients [8]. The use of PTX dimer nanoparticles has also been proposed as a powerful alternative for increasing the solubility of the drug, as well as the PTX content of the delivery system (up to 85%), which is very interesting [67].

Table 2 summarizes the main aspects of approved PTX-alternatives to Taxol^®^. The table shows the composition, the company that supplies the drug, and the place where it is approved. It also indicates the type of target cancer and the common and maximum authorized dose (MTD) (the common dose and MTD for Taxol^®^ are 175 and 240 mg/m^2^, respectively) [8]. Finally, the advantages of its use over Taxol^®^ are summarized.

One of the greatest challenges in the fight against cancer is the development of drugs that act specifically on tumor cells, that is, targeted drugs. Regarding PTX, several tumor-targeted delivery systems have been developed, where PTX is coupled with different molecules, such as peptides, proteins, antibodies, and polymers [59,68,69,70,71]. Most of them are formulated as prodrugs and their release takes place in response to stimuli, such as an acidic environment, an elevated reactive-oxygen species (ROS) level, and an increased glutathione (GSH) level, typical of tumor tissues, which considerably increases their effectiveness [46,72,73,74,75,76,77,78,79,80,81]. In this sense, a recent study carried out by Mu et al. developed acid-sensitive polyethylenglycolylated (PEGylated) acetone-based acyclic-ketal-linked-PTX nanoparticles (PKP NPs). They evaluated the effect of three different nanoparticles (with different PEG lengths) and compared them with the effect of Taxol^®^ on human breast cancer cells, human ovarian cancer cells, and human pulmonary carcinoma cells. Although the cytotoxicity of Taxol^®^ was higher, the new formulations showed a much lower hemolytic activity and a higher MTD. Regarding pharmacokinetics, Taxol^®^ exhibited a short t_1/2_ (1.04 h), while PKP NPs displayed longer blood circulation and t_1/2_. As a consequence, the area under the concentration-time curve (AUC_0∞_) was higher for the new formulations than for Taxol^®^. Finally, the biodistribution was evaluated in human ovarian carcinoma cells treated with PKP NPs or Taxol^®^. PKP NPs demonstrated higher accumulation in tumor cells and an increased concentration of free PTX at 12 h [82].

In recent years, exosomes have become an interesting alternative to natural nanoparticles for drug delivery, especially due to their low immunogenicity and high biocompatibility [83]. Therefore, PTX-loaded exosomes have showed an increased cytotoxicity in PTX-resistant cells [84]. Moreover, a recent study has demonstrated a targeted PTX delivery system combining an exosome carrier and a PEG derivative, representing a novel delivery platform for anticancer therapy [85]. In this study, Kim et al., incorporated an aminoethyl anisamide-polyethylene glycol (AA-PEG) vector to PTX-loaded exosomes (AA-PEG-exoPTX) to specifically target the sigma receptor, which is overexpressed in lung tumor cells. In total, 94.4% of AA-PEG-exoPTX were co-localized with lung metastases animals, while no AA-PEG-exoPTX were found in healthy animals. Moreover, an injection of AA-exoPTX nanoparticles caused a strong suppression of metastases growth and greater survival time, with respect to Taxol^®^.

Regarding the administration route of the drug, traditional formulations have been administered intravenously due to the low oral bioavailability of PTX [86]. However, the presence of organic solvents to increase Taxol^®^ and derivates’ solubility may have adverse effects. Therefore, alternatives have been developed to increase the oral bioavailability, such as formulations with PTX conjugated to chitosan, lipid derivates, nanocochleates, hyaluronic acid-octadecylamine micelles, and oil-based nanocarriers [54,86,87,88,89,90,91]. PTX loaded in milk-derived exosomes has also shown a high oral bioavailability and low toxicity as a system to maximize oral chemotherapy [92]. On the other hand, the PTX oral bioavailability has been demonstrated to be enhanced by the coadministration of P-gp inhibitors. P-glycoprotein (P-gp) is a membrane multidrug transporter responsible for the extrusion of PTX from tumor cells, which considerably reduces its bioavailability and contributes to creating a resistance against this and other drugs [93,94]. Several inhibitors (first, second, and third generation) have been shown to increase the oral bioavailability. Some of the most studied are cyclosporin A and its analogues, and the inhibitor Tariquidar [95,96]. However, despite the progress that has been made, these inhibitors continue to show limitations, especially in regard to cytotoxicity [97]. In this sense, in a recent study carried out by Chen et al., chitosan polymeric micelles providing an oral supply of paclitaxel have been developed. Moreover, a multi-functional chitosan copolymer has been designed to increase bioadhesion and inhibit P-gp efflux. Altogether, the results showed that PTX-micelles altered pharmacokinetics and increased the therapeutic effect of paclitaxel. The bioavailability of micelles was increased 3.80-fold with respect to PTX and, as a consequence, the anti-tumor efficacy was also enhanced [98].

### 1.4. Taxol^®^ Antitumoral Mechanism

Cancer encompasses a group of diseases that affect different tissues, all of which are characterized by the uncontrolled growth of cells that can invade other areas of the body. In 2018, the Word Health Organization (WHO) stated that one in five men and one in six women worldwide will develop cancer during their lifetime, and that one in eight men and one in eleven women will die of the disease. Based on this statistic, it seems reasonable that one of the great challenges of humanity at present is to find definitive treatments to combat different cancers. In 1998, the FDA officially approved Taxol^®^ as a potential anticancer drug and, since then, it has become a widely employed anticancer drug [107].

**Figure 2:**
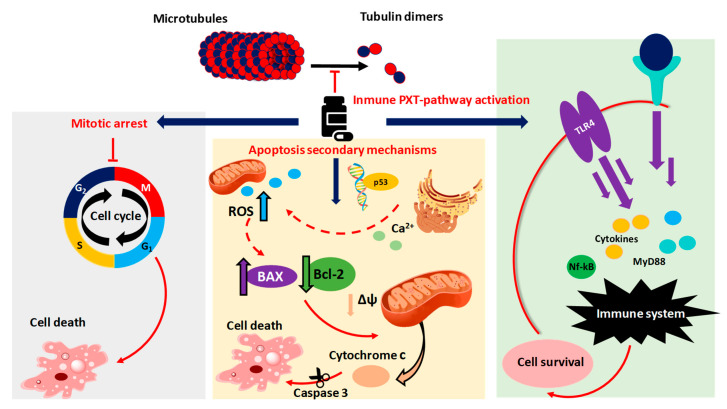
Proposed paclitaxel mechanisms of action in cancer cells. Discontinuous lines indicate suggested mechanisms.

Unlike most antitumor drugs, which are aimed at damaging DNA or RNA, the main mechanism of Taxol^®^ is the promotion of cellular death through binding to tubulin and inhibiting the disassembly of microtubules [108,109]. Therefore, Taxol^®^ is one of the so-called Microtubule Targeting Agents (MTAs) [4]. Microtubules are tubulin heterodimers involved in many important cellular processes [110]. The production of tubulin and the microtubules assembly occurs during the G2 phase of the cell cycle. MTAs can be classified into microtubule stabilizing agents, such as Taxol^®^, and destabilizing agents, such as *vinca* alkaloids, which bind to the α/β tubulin in order to disassemble microtubules. Therefore, although both cause cell death and are widely used anti-cancer agents, they have opposite mechanisms of action [111]. In fact, cells exposed to PTX are detained in the G2/M-phase and finally, the non-progression of the cell cycle leads to its death (Figure 2) [12,112]. However, it is important to underline that cells can carry out a premature mitotic exit—known as “mitotic slippage”—which is the major mechanism of escape from MTAs, limiting the efficacy of these drugs [113].

Despite the fact that mitotic arrest is the main mechanism of PTX, it has been assumed that it also causes cell death in tumors through the activation of metabolic apoptosis (Figure 2). Therefore, although it is not entirely clear how Taxol^®^ induces cellular apoptosis, it has been suggested that apoptosis is related to the activation of transcription factor p53, associated with the progression of numerous human tumors [114,115]. According to a recent study, PTX produces an increase in ROS and an overexpression of the genes and proteins related to stress of the endoplasmic reticulum (ER) in osteosarcoma cells [116]. However, it remains to be shown whether the stress on the endoplasmic reticulum derives from gene dysregulation, caused by p53 activation. On the other hand, it has been suggested that damage to the ER may cause a release of Ca^2+^, which provokes Ca^2+^ overload and mitochondrial damage, leading to an increase in ROS production [117,118]. Furthermore, another recent study has demonstrated that, in canine mammary gland tumor cells, PTX induces a downexpression of the anti-apoptotic protein B-cell Leukemia 2 (Bcl-2) and an overexpression of the pro-apoptotic protein Bcl-2-associated X protein (BAX) [114,115,119,120]. These alterations are responsible for the triggering of mitochondrial apoptosis through disruption of the mitochondrial membrane potential (MMP) and the consequent release of cytochrome C from mitochondria into the cytoplasm and cleavage of the caspase-3 protein [121,122] (Figure 2). However, it is not entirely clear whether mitochondrial apoptosis is directly induced by an increase of ROS [114].

Recently, the autophagy protein 5 (ATG5) has been demonstrated to be involved in the autophagic response induced by PTX [123]. Today, there is a great controversy about the role of autophagy in the death and survival of tumor cells. Although some research assures that autophagy is one of the mechanisms of antitumor agents, such as Taxol^®^, for killing cancer cells, others suggest that it is a cell survival mechanism [124,125,126,127]. Therefore, it is necessary to continue conducting research to clarify how autophagy influences the development of cancer and to develop new therapeutic treatments [128].

PTX has been widely shown to cause dysregulation of the toll-like receptor 4 (TLR4) inflammation pathway. This pathway, which plays a fundamental role in the defense of cells under normal conditions, is also associated with a decrease in the antitumor effect of PTX [120,129,130,131,132]. The presence of inflammation mediators in PTX-treated cells has led researchers to think that treatment with this drug induces a cascade (PTX pathway), resulting in the survival of PTX-treated cells and the development of resistance to the drug (Figure 2). In 2013, Rajput et al. suggested, for the first time, that the PTX-pathway is induced after TLR4 dysregulation in human breast cancer cells [133]. TLRs recognize pathogen-associated molecular patterns (PAMPs) to produce immune responses, such as the secretion of inflammatory mediators [134]. Regarding TLR4, its dysregulation triggers MyD88-dependent pathways, which result in the activation of several pro-oncogenic signals, including the nuclear factor kappa-light-chain-enhancer of activated B cells (NF-κB), and inflammatory cytokine production [113,135,136,137,138,139]. The discovery of the relationship between PTX treatment and TLR4 dysregulation led to a new way to maintain the effectiveness of Taxol^®^ and to minimize the resistance shown by tumor cells. The new goal was to block or attenuate the negative effects of TLR4-mediated PTX resistance. Conversely, a recently published work showed that the PTX-pathway through TLR4 could contribute to the antitumor effect of PTX by reprogramming macrophages to phase M1, which would reactivate the immune response against cancer [140].

In addition to the TLR-4 cascade, a recent study pointed out the dysregulation of the NLRP3 inflammasome by PTX. The NLRP3 inflammasome is a component of the innate immune system, releasing proinflammatory cytokines IL-1b and IL-18. However, the effect of the NLRP3 inflammasome on the cytotoxic Taxol^®^ power remains unclear [141]. Moreover, it has been suggested that PTX treatment is not directly responsible for mitochondrial damages, but that they are mediated by the NLPR3 pathway [118].

## 2. The Fight against Drug Resistance

In recent years, many studies have focused on the effect of Taxol^®^ in order to improve results among patients. Even though PTX is one of the most effective and frequently used drugs for the treatment of different cancers, its efficiency is limited. Therefore, one of the major drawbacks of Taxol^®^ and derivatives is the acquisition of resistance in many of the patients treated. Tumor suppressor genes (TSGs) are essential genes related to cell division, apoptosis, and DNA repair. Normally, these TSGs prevent abnormal cells from surviving. However, when these genes are inactivated or their expression is reduced, the abnormal cells may grow uncontrollably, thus leading to cancer formation; considering this, they could be important mediators of drug sensitivity. In order to increase the knowledge about PTX resistance, Xu et al. identified 22 TSGs involved in PTX resistance in 2016, representing an excellent inflection point for developing new strategies to reverse chemotherapy resistance [142]. In the same line, Wu et al. carried out a transcriptomic study of PTX-resistant esophageal squamous cell carcinoma (ESCC), in order to identify potential genes and pathways responsible for the PTX resistance. They concluded that carfilzomib—a proteasome inhibitor—could attenuate paclitaxel resistance through activating hypoxia-inducible factor 1 (HIF-1) signaling [143]. Resistance to paclitaxel has been related to different proteins, such as keratin 17 (KRT17) in cervical cancer cells, which may increase cell migration and PTX survival, or fibronectin type III domain-containing protein 5 (FNDC5), which could promote paclitaxel sensitivity by inhibiting NF-κB/MDR1 signaling in NSCLC [144,145].

The multidrug resistance-associated protein 1 (MDR1) and the P-gp have also been demonstrated to be associated with the acquisition of paclitaxel resistance. P-gp and MDR1 are members of the ATP-binding cassette (ABC) family, whose role is to mediate MDR through the efflux of drugs out of the cell, preventing the cellular accumulation of anti-tumor drugs [94]. Paclitaxel is a high-affinity substrate for P-gp and MDR1, which causes a decrease in the bioavailability and induces PTX-resistance [146,147]. Therefore, one of the main strategies for combatting PTX-resistance is the use of MDR1 and P-gp inhibitors [148]. In this sense, recent studies have shown that resistance can be decreased in ovarian cancer with Myricetin or G-quadruplex oligonucleotides, which downregulate (MDR)-1/P-glycoprotein and induce cell apoptosis [149,150].

The receptor tyrosine kinase (RTK) family, which includes EGFR, erbB2, erbB3, and erbB4 (also known as HER2, HER3, and HER4), is an important family of receptors involved in tumorigenesis which have been widely studied as therapeutic targets. The role of RTKs is to activate a multiplicity of intracellular pathways through ATP-mediated auto-phosphorylation. Therefore, inhibitors of receptor tyrosine kinases (TKIs) block their kinase activity, thus avoiding the activation of RTK pathways [151,152]. TKIs have demonstrated an excellent clinical efficacy, although disabling adverse effects and the development of resistance have also been associated with their use. In this way, many studies are being carried out to improve the effects of Taxol^®^ and other PTX derivatives, in order to combat the tumor cell resistance. Mucin 1 (MUC-1), which is a transmembrane glycoprotein overexpressed in cervix tumor cells, has been found to be involved in PTX chemoresistance in an EGFR-dependent manner. The treatment of cervical cancer cells with the EGFR-TKI Erlotinib has shown it to be an adequate alternative when PTX resistance has been acquired [153]. Another study, carried out by Gupta et al., demonstrated that PTX-resistant metastatic breast cells exhibited an increased expression of erbB2 and β-catenin pathways. Penfluridol (PFL) treatment suppressed this erb2 and β-catenin expression, opening up new avenues for PFL/PTX treatment [154]. However, despite these efforts, Aldonza et al. have recently reported that cancer cells with an acquired PTX resistance often develop a tolerance to TKIs as a consequence of PTX treatment [155].

Forkhead protein 1 (FoxM1) has also been correlated with PTX resistance in human pancreatic cancer, through a pathway involving the overexpression of Prohibitin 1 (PHB1) and activation of the FoxM1/PHB1/RAF-MEK-ERK pathway [156]. Moreover, it has recently been demonstrated that Aurora kinase A stabilizes FoxM1 in PTX-resistant breast tumor cells [56]. Therefore, targeting FoxM1 or Aurora kinase A could provide promising alternatives to decrease the resistance to Taxol^®^ developed by many cells. Otherwise, octreotide, which is a somatostatin analogue (SSTA) able to inhibit tumor proliferation through binding to the somatostatin receptor (SSTR), might reserve PTX-resistance. Therefore, it has been suggested that the paclitaxel-octreotide conjugate in ovarian tumor cells decreases PTX chemoresistance through the p38 Mitogen-Activated Protein Kinase (MAPK) signaling pathway [157,158].

**Figure 3:**
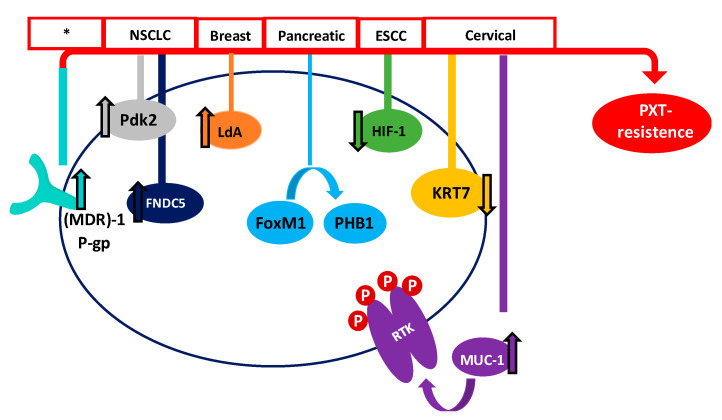
Main proposed mechanisms of PTX survival in different cancers. Red boxes show the type of cancer where the mechanism has been proposed (* many types of cancer).

On the other hand, metabolic alterations have been shown to play an important role in the sensitivity of cancer cells to anticancer agents [159]. Therefore, the increased expression of lactate dehydrogenase-A plays an important role in Taxol^®^ resistance in human breast cancer cells. In this sense, treating cells with a combination of Taxol^®^ and oxamate (a non-competitive inhibitor of the enzyme lactate dehydrogenase) resulted in a synergistical inhibitory effect on Taxol-resistant breast cancer cells [160]. More recently, Sun et al. have shown that the high PTX resistance demonstrated by lung cancer cells is associated with an increased expression of pyruvate dehydrogenase kinase-2 (Pdk2), which is a key regulator of glycolysis and oxidative phosphorylation [161]. Figure 3 summarizes some of the proposed mechanisms, but definitely does not cover them all.

It is also worth highlighting the alterations in the dynamics of microtubules and tubulin, which may influence the binding of the anti-tumor agent [162]. Therefore, an altered expression of tubulin isotypes is considered a prognostic marker of chemotherapy drug resistance and survival [163,164]. In fact, many studies have shown the expression of different α and β tubulin isotypes in cancer cells. Moreover, the isotype expression depends on the type of tumor [163]. Therefore, high levels of αIIb tubulin in hepatocellular carcinoma; βI in ovarian cancer and NSCLC adenocarcinoma; βII in lung adenocarcinoma; βIII in colon cancer, uterine serous carcinoma, and melanoma; and βV in NSCLC, are associated with a resistance to PTX [165,166,167,168,169,170,171]. On the other hand, a relationship between mutations and post-translational modifications found in β-tubulin and resistance to anti-tumor agents has also been suggested, although further research is necessary in this area [172,173,174].

Other ways to combat the resistance of cancer cells to existing drugs include the search for new agents with cytotoxic properties and the anticipation of the effectiveness of the treatments in each patient. Therefore, the search for new natural compounds with antitumor activity and the co-release of PTX with other natural compounds such as flavonoids or polyphenols also appears to be a promising alternative [43,175,176,177]. Another way to improve current cancer (and other disease) treatments is to predict the effectiveness for each patient before scheduling their treatment. In this way, Ben-Hamo et al. observed that the sequence of the Bcl-2 anti-apoptotic protein might act as a PTX chemoresistance indicator. They found that patients showing a single-nucleotide variant in the Bcl-2 sequence may be more resistant to PTX. Therefore, previous knowledge of Bcl-2 genomic sequences could help to improve patient survival [178]. In a similar way, the methylome and miRNome of breast cancer cells could be used as molecular signatures to predict the paclitaxel response [155]. Moreover, recently, it has been demonstrated that peripheral neuropathy (PN), which is a common adverse effect induced by PTX in patients with breast cancer, could be predicted by miRNA451a expression [179].

## 3. RNA-Based Therapies

In the last decade, several researches have focused on noncoding RNA and its role in gene regulation. Most noncoding RNA is processed to generate small RNA such as miRNA, or long noncoding RNA (lncRNA). Due to their involvement in gene regulation, both miRNAs and lnRNAs have been related to diseases such as cancer, becoming hopeful therapeutic targets and biomarkers, as well as potent agents to fight against drug resistance [180,181].

Regarding PTX, and especially resistance generated by tumor cells, several recent studies have demonstrated the relationship between miRNAs and lnRNAs, and PTX sensitivity [178,179,182,183,184]. miRNAs 16, 200c, 203, 34a, 107, 124, 421, 18a, and 29c have been shown to be differentially expressed in PTX-resistant tumor cells, and consequently, their protein targets were dysregulated. The treatment of tumor cells modulating miRNAs decreased the PTX resistance [28,185,186,187,188,189,190,191]. Moreover, the treatment of gastric cancer cells combining Taxol with miR-200a and FH535 has been shown to increase the apoptotic effect [192]. In a similar way, miR-193a replacement combined with Taxol^®^ chemotherapy improved the response in colorectal cancer cells [193].

In a similar way, different lncRNAs have also been related to PTX resistance. The lncRNA eosinophil granule ontogeny transcript (EGOT), the lnRNA Fer-1-like family member 4 (FER1L4), the lncRNA KB-1471A8.2, the lncRNA NONHSAT141924, and the lncRNA intergenic non-protein-coding RNA p53-induced transcript (LINC-PINT) have been shown to be up- or downregulated in different PTX-resistant cancer cells. As a consequence, chemoresistance could be decreased by modulating lnRNA levels [194,195,196,197,198]. Moreover, recent studies suggest a regulation of lncRNAs through miRNAs, and underline the importance of a lncRNA-miRNA-mRNA network for regulating gene expression and cancer [199,200,201] (Table 3). LncRNA Urothelial Carcinoma Associated 1 (UCA1) is involved in PTX ovarian cancer cell resistance through a miRNA129-ABCB1 (ATP Binding Cassette Subfamily B Member 1) axis [202]. Long Intergenic Non-Coding RNA 1118 (LINC01118) is overexpressed in ovarian- and paclitaxel-cisplatin-resistant tumor cells and this upregulation has been related to miRNA134-ABCB1 [203]. Upregulation of the lnRNA succinate dehydrogenase complex flavoprotein subunit A pseudogene 1 (lncRNA SDHAP1) in tumor ovarian cells contributes to PTX resistance through miR-4465-eukaryotic translation initiation factor 4 gamma 2 (EIF4G2) [204]. Long non-coding RNA FTH1P3 activates PTX resistance in breast cancer through miR-206/ABCB1 [205], and lncRNA colon cancer-associated transcript 1 (CCAT1) is overexpressed in prostate cancer, with knockout increasing the sensitivity to PTX through miR-24-3p and fascin1 (FSCN1) [206]. The resistance to PTX developed by some ovarian tumor cells has also been related to the presence of miRNA21-derived cancer-associated adipocytes (CAAs) and fibroblasts (CAFs) from exosomes. Therefore, this resistance could be combated by inhibiting the transfer of miRNA21 [207,208].

Table 3 summarizes some examples of non-coding RNAs related to PTX resistance. However, it is important to emphasize that the knowledge about the role of noncoding RNA in cancer is limited and it is necessary to increase our knowledge in order to improve anti-tumor treatments and combat the resistance created by cancer cells.

## 4. Conclusions and Future Challenges

The fight against cancer is and, for decades to come, will probably continue to be, one of the main human battles. In this way, although the effectiveness of new drugs is increasing, cancer continues to be very lethal. Taxol^®^, along with the rest of the compounds derived from PTX, have been, and continue to be, very useful in the fight against this disease. However, the two main disadvantages of its use remain unresolved: Its production is both expensive and unsustainable, and the mechanisms by which tumor cells develop resistance to it are not yet fully clear.

The production of PTX by microbial fermentation is the most promising alternative for competing with chemical synthesis and extraction from plants. However, a greater understanding of microbial metabolism and the development of better genetic engineering techniques are still necessary. The main challenge is the effective oxygenation of non-oxygenated intermediates, such as taxadiene, in order to achieve the complete synthesis of Taxol^®^. Endophytic fungi-based production is also a hopeful emerging alternative to traditional and poor environmentally friendly methods. However, again it is necessary to increase the metabolic knowledge associated with these processes in order to improve production and ensure that it is maintained after several subcultures.

Regarding the fight against resistance developed by many of the patients treated with PTX, the research currently underway is very intense. Much progress has been made towards understanding the PTX pathway, although it still remains unclear. According to the conclusions reached to date, this resistance may be due to the different pathways activated by the drug, which makes it more difficult to combat. To achieve this, it is necessary to continue developing research in this field and increase our knowledge, in order to improve the efficacy of Taxol^®^, as well as other known anticancer agents.

## Figures and Tables

**Table 1:** Main processes developed to obtain Taxol^®^ intermediates through microbial fermentation.

Product	Microbial Host	Maximum Yield	Reference
Taxadiene	*E. coli*	1000 mg/L	[33]
	*S. cerevisiae*	72.8 mg/L	[35]
	*B. subtilis*	17.8 mg/L	[36]
Oxygenated taxanes	*E. coli*	570 mg/L	[37]

**Table 2:** Main characteristics of the approved alternative formulations to Taxol^®^. mPEG-PDLLA: Monomethoxy-poly (ethylene glycol)-block-poly(d,l-lactide); PVP-b-PNIPAAM: Poly-(vinylpyrrolidone)-b–poly-(*N*-isopropyl acryl-amide); N-tr-Lc: *N*-(all-trans-retinoyl)-l-cysteic acid; and N-13cr-Lc: *N*-(13-cis-retinoyl)-l-cysteic acid methyl ester.

Commercial Name	Formulation	Company	Delivery System (Nanoparticle Diameter)	Status	Cancer Type tXarget	Advantages	Common Dose/MTD (mg/m^2^)	Ref.
Abraxane^®^ (nab-PTX)	Paclitaxel and albumin.	Celgene 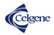 Abraxis 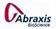	Albumin-bound paclitaxel nanoparticles (130 nm)	Approved internationally (FDA in 2005, EMA in 2008).	Breast cancer, NSCLC, and pancreatic cancer.	Lower toxicity. High MTD.	260/300	[8,12,99,100]
Cynviloq™ (Genexol-PM^®^)	Paclitaxel and mPEG-PDLLA.	Samyang 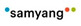 Nantpharma 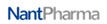	Polymeric micelles (25 nm)	Approved in South Korea in 2007.	Breast cancer, NSCLC, ovarian cancer, pancreatic cancer, and bladder cancer.	Lower toxicity. The absence of albumin reduces the risk of microbial growth. High MTD.	260/390	[8,12,52,101,102]
Lipusu^®^	Paclitaxel, lecithin, and cholesterol.	Luye Pharma 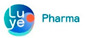	Liposome (400 nm)	Approved in China in 2006.	Ovarian cancer and NSCLC (approved). Breast cancer (clinical trial).	Lower toxicity.	175/no data	[8,12,52,103]
PICN	Paclitaxel, polyvinyl-pyrrolidone, cholesteryl sulfate, and caprylic.	Sun Pharma 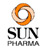	Polymeric lipid-nanoparticles (100 nm)	Approved in India in 2014.	Breast cancer (approved). Ovarian cancer and bladder cancer (clinical trial).	Lower toxicity. High MTD.	260, 95/325	[8,12,52,102]
Nanoxel^®^	Paclitaxel and PVP-b-PNIPAAM.	Dabur 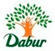	Polymeric micelles (80–100 nm)	Approved in India in 2008.	Breast cancer, NSCLC, ovarian cancer, and AIDS-related Kaposi’s sarcoma (approved).	The carrier is pH-sensitive (tumor targeted drug). High MTD. Lower toxicity.	300/375	[12,52,104,105]
DHP-107 (Liporaxel^®^)	Paclitaxel, monoolein, tricaprylin, and Tween 80.	Daehwa 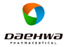	Emulsion (oral administration)	Approved in South Korea in 2016.	Gastric cancer.	Oral administration. High MTD.	200/600	[8,12,87]
(Apealea^®^) Paclical^®^	Paclitaxel, N-tr-Lc methyl ester, and N.13cr-Lc methyl ester.	Oasmia 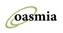	Polymeric micelles (20–60 nm)	Approved in Russian Federation in 2015.	Ovarian cancer (approved).	The carrier is rapidly metabolized.	260/250	[8,12,52,106]

**Table 3:** miRNAs and long noncoding RNAs (lncRNAs) involved in PTX resistance.

RNA	Metabolic Target	Differential Expression Found in PTX-Resistant Cells	Reference
200c	Cathepsin L (CTSL)	Downregulation	[186]
203	Salt-inducible kinase 2 (SIK2)	Downregulation	[28]
18a	Endoribonuclease Dicer	Upregulated	[191]
16	IκB kinase β (IKBKB)	Downregulation	[209]
107	Antiapoptotic factor Bcl-w	Downregulation	[187]
34a	E2F transcription factor 5 (E2F5)	Downregulation	[185]
124	Monocarboxylic acid solute transporter 1 (MCT1)	Downregulated	[188]
421	Kelch-like ECH-associated protein 1 (KEAP1)	Upregulation	[190]
29c	Upregulation of integrin beta-1 (ITGB1).	Downregulation	[189]
EGOT	Inositol 1,4,5-Trisphosphate Receptor Type 1 (ITPR1)	Downregulated	[194]
UCA1	miRNA129/ABCB1	Upregulated	[202]
FER1L4	MAPK	Downregulated	[195]
LINC01118	miRNA134/ABCB1	Upregulated	[203]
KB-1471A8.2	Cyclin-dependent kinase 4 (CDK4)	Downregulated	[196]
SDHAP1	miR-4465/EIF4G2	Upregulated	[204]
NONHSAT141924	p-CREB/Bcl-2	Upregulated	[197]
LINC-PINT	NONO (a non-POU-domain-containing, octamer-binding protein)	Downregulated	[198]
FTH1P3	miR-206/ABCB1	Upregulated	[205]
CCAT1	miR-24-3p/FSCN1	Upregulated	[206]

## Abbreviations

PTX	Paclitaxel
NCI	National Cancer Institute
*T. brevifolia*	*Taxus brevifolia*
BMS	Bristol-Myers Squibb
FDA	Food and Drug Administration
EMA	European Medicines Agency
NSCLC	Non-small-cell lung carcinoma
PDAC	Pancreatic ductal adenocarcinoma
MVA	Mevalonate
MEP	methylerythritol phosphate
IPP	Isopentenyl pyrophosphate
DMAPP	Dimethylalyl pyrophosphate
taxadiene	Taxa-4(5),11(12)-diene
IDI	Isopentenyldiphosphate isomerase
GPPS	Geranylpyrophosphate synthase
GGPPS	Geranylgeranylpyrophosphate synthase
10-DAB	10-deacetyl baccatin III
*E. coli*	*Escherichia coli*
*S. cerevisiae*	*Sacharomyces cerevisiae*
*B. subtillis*	*Bacillus subtilis*
CrEL	Cremophor EL
MTD	Maximum authorized dose
ROS	Reactive-oxygen species
GSH	Glutathione
PEG	polyethylenglycoly
AUC	Area under the concentration–time curve
P-gp	P-glycoprotein
mPEG-PDLLA	Monomethoxy-poly (ethylene glycol)-block-poly(d,l-lactide)
PVP-b-PNIPAAM	poly-(vinylpyrrolidone)-b–poly-(*N*-isopropyl acryl-amide)
WHO	Word Health Organization
MTA	Microtubule Targeting Agent
ER	Endoplasmic reticulum
Bcl-2	B-cell Leukemia 2
BAX	Bcl-2-associated X protein
MMP	Mitochondrial membrane potential
ATG5	Autophagy protein 5
TLR4	Toll-like receptor 4
PAMPs	Pathogen-associated molecular patterns
HIF-1	Hypoxia-inducible factor 1
KRT17	keratin 17
FNDC5	Fibronectin type III domain-containing protein 5
MDR1	Multidrug resistance-associated protein 1
ABC	ATP-binding cassette
RTK	Receptor tyrosine kinase
TKIs	Inhibitors of receptor tyrosine kinases
MUC-1	Mucin 1
PFL	Penfluridol
FoxM1	Forkhead protein 1
PHB1	Prohibitin 1
SSTA	Somatostatin analogue
SSTR	Somatostatin receptor
MAPK	Mitogen-Activated Protein Kinase
LdA	Lactate dehydrogenase
Pdk2	Pyruvate dehydrogenase kinase 2
PN	Peripheral neuropathy (PN)
lncRNA	Long noncoding RNA
EGOT	Eosinophil granule ontogeny transcript
FER1L4	Fer-1-like family member 4
LINC-PINT	lncRNA intergenic non-protein-coding RNA p53-induced transcript
UCA1	Urothelial Carcinoma-Associated 1
ABCB1	ATP Binding Cassette Subfamily B Member
LINC01118	Long Intergenic Non-Coding RNA 1118
lncRNA SDHAP1	LnRNA succinate dehydrogenase complex flavoprotein subunit A pseudogene 1
EIF4G2	Eukaryotic translation initiation factor 4 gamma 2
CCAT1	Colon cancer-associated transcript 1
FSCN1	Fascin1
CAAs	Cancer-associated adipocytes
CAFs	Cancer-associated fibroblasts
(CTSL	Cathepsin L
SIK2	Salt-inducible kinase 2
IKBKB	IκB kinase β
E2F5	E2F transcription factor 5
MCT1	Monocarboxylic acid solute transporter 1
KEAP1	Kelch-like ECH-associated protein 1
ITGB1	Upregulation of integrin beta-1
ITPR1	Inositol 1,4,5-Trisphosphate Receptor Type 1
